# Characterization of the novel broad-spectrum lytic phage Phage_Pae01 and its antibiofilm efficacy against *Pseudomonas aeruginosa*

**DOI:** 10.3389/fmicb.2024.1386830

**Published:** 2024-07-17

**Authors:** Zhixin Shi, Xin Hong, Zexuan Li, Meijuan Zhang, Jun Zhou, Zhe Zhao, Shengfeng Qiu, Genyan Liu

**Affiliations:** ^1^Department of Laboratory Medicine, The First Affiliated Hospital With Nanjing Medical University, Nanjing, China; ^2^National Key Clinical Department of Laboratory Medicine, The First Affiliated Hospital With Nanjing Medical University, Nanjing, China; ^3^College of Oceanography, Hohai University, Nanjing, China

**Keywords:** *Pseudomonas aeruginosa*, bacteriophage, phage therapy, multidrug resistance, biofilm, gentamicin

## Abstract

**Introduction:**

*Pseudomonas aeruginosa* is present throughout nature and is a common opportunistic pathogen in the human body. Carbapenem antibiotics are typically utilized as a last resort in the clinical treatment of multidrug-resistant infections caused by *P. aeruginosa*. The increase in carbapenem-resistant *P. aeruginosa* poses an immense challenge for the treatment of these infections. Bacteriophages have the potential to be used as antimicrobial agents for treating antibiotic-resistant bacteria.

**Methods and Results:**

In this study, a new virulent P. aeruginosa phage, Phage_Pae01, was isolated from hospital sewage and shown to have broad-spectrum antibacterial activity against clinical *P. aeruginosa* isolates (83.6%). These clinical strains included multidrug-resistant *P. aeruginosa* and carbapenem-resistant *P. aeruginosa*. Transmission electron microscopy revealed that the phage possessed an icosahedral head of approximately 80 nm and a long tail about 110  m, indicating that it belongs to the Myoviridae family of the order *Caudovirales*. Biological characteristic analysis revealed that Phage_Pae01 could maintain stable activity in the temperature range of 4~ 60°C and pH range of 4 ~ 10. According to the *in vitro* lysis kinetics of the phage, Phage_Pae01 demonstrated strong antibacterial activity. The optimal multiplicity of infection was 0.01. The genome of Phage_Pae01 has a total length of 93,182 bp and contains 176 open reading frames (ORFs). The phage genome does not contain genes related to virulence or antibiotic resistance. In addition, Phage_Pae01 effectively prevented the formation of biofilms and eliminated established biofilms. When Phage_Pae01 was combined with gentamicin, it significantly disrupted established *P. aeruginosa* biofilms.

**Conclusion:**

We identified a novel *P. aeruginosa* phage and demonstrated its effective antimicrobial properties against *P. aeruginosa* in both the floating and biofilm states. These findings offer a promising approach for the treatment of drug-resistant bacterial infections in clinical settings.

## 1 Introduction

*Pseudomonas aeruginosa* is a gram-negative bacillus that exists widely in natural environments such as soil, air, and lakes, as well as on the skin, in respiratory tract, and in intestines of healthy individuals. *P. aeruginosa* is also one of the most common opportunistic pathogens that causes nosocomial infections (Wang et al., 2021). Additionally, *P. aeruginosa* is prevalent in respiratory tract infections, urinary tract infections, cystic fibrosis, septicaemia patients, and individuals with compromised immune function due to long-term chemotherapy or the use of immunosuppressants (Angeletti et al., 2018). *P. aeruginosa* has a strong capacity for biofilm formation. *P. aeruginosa* biofilms play an important role in the development of chronic bacterial infectious diseases such as cystic fibrosis, subacute bacterial endocarditis, and periodontitis (Hassett et al., 2009). Furthermore, biofilms are often formed on armamentarium and interventional therapy devices, such as catheters, vascular catheters, and tracheal intubation devices. The resistance of biofilms to antibiotics and other antimicrobial agents contributes to treatment failure in some chronic and refractory diseases (Pang et al., 2019; Thi et al., 2020).

In recent years, the overuse of antibiotics in the field of human medicine has precipitated a rapid increase in the prevalence of antibiotic-resistant pathogens (Kao et al., 2016). The World Health Organization considers infections caused by multidrug-resistant bacteria to be a major public health concern (Tacconelli et al., 2018). The prevalence of carbapenem-resistant *P. aeruginosa* (CRPA) in clinical settings has significantly increased as a result of the widespread and improper use of various antibiotics, including carbapenems. This situation undoubtedly brings tremendous burdens and challenges to the global health care landscape and clinical anti-infective treatment (Tacconelli et al., 2018). Consequently, there is an urgent need to explore new treatment methods beyond traditional antibiotic therapy. Bacteriophages, which are viruses capable of infecting bacteria and are widely present in the environment, have attracted increased amounts of attention since the early 20th century (Chegini et al., 2020). Nevertheless, the convenience, cost-effectiveness, and rapidly development of antibiotics, coupled with the limitations of early bacteriophage therapy, have led to a gradual decrease in interest in bacteriophage therapy. The global dissemination of drug-resistant strains and the lengthy and costly research and development process for new antimicrobial compounds have revived interest in bacteriophage therapy. Compared to other antibacterial options, bacteriophages have the advantages of widespread distribution, strong specificity, few side effects, and the ability to self-amplify at the site of infection, which further enhances their efficacy as antibacterial agents (Lin et al., 2017). Consequently, these characteristics make bacteriophages an ideal choice for combating bacterial infections (Strathdee et al., 2023). Additionally, the increasing number of successful clinical cases of bacteriophage therapy use for the treatment of multidrug-resistant bacterial infections in recent years has provided evidence of the potential promise of bacteriophage therapy as an effective antibacterial strategy (Chan et al., 2019; Tkhilaishvili et al., 2020).

In this study, we isolated and identified a novel lytic phage, Phage_Pae01, which has a broad host range, including CR-PA and MDR-PA. The biological characteristics and genome sequence of Phage_Pae01 were further analysed. The ability of Phage_Pae01 to destroy *P. aeruginosa* biofilms *in vitro* and the ability of the phage alone or in combination with gentamicin to eliminate *P. aeruginosa* biofilms were studied. All the evidence suggests that Phage_Pae01 has the potential to serve as a good candidate for phage therapy; this phage offers a new approach for the prevention and treatment of *P. aeruginosa*-related infections in the future.

## 2 Materials and methods

### 2.1 Bacterial strains and growth conditions

The host bacterium Pa021 was isolated from the sewage treatment centre of the Jiangsu Women and Children Health Hospital and identified by VITEK MS (bioMérieux, SA). *P. aeruginosa* used in phage host range experiments was collected from Jiangsu Women and Children Health Hospital and Jiangsu Province Hospital. The source and antibiotic resistance information of all strains can be found in Supplementary Tables S1, S2. All strains were thawed from the −80°C freezer and streaked onto blood agar plates. Single colonies were selected in sterile Luria–Bertani (LB OXOID, UK) liquid media to prepare the bacterial solutions.

### 2.2 Phage isolation, purification, and propagation

The host bacterium Pa021 was cultured at 37°C with constant-temperature shaking at 180 rpm for 6 h to obtain the bacterial solution. A sample of 500 ml of untreated sewage was collected from Jiangsu Women and Children Health Hospital. The sample was then centrifuged at 8,000 rpm for 10 min to remove precipitated particles and filtered through a 0.22 μm filter (Millipore, USA). The treated sewage sample was mixed with an equal volume of sterile LB liquid media and inoculated with 10 ml of solution with the host bacterium Pa021. The mixed sample was cultured at 37°C with shaking at 180 rpm for 24 h. Next, the culture fluid was collected and centrifuged at 8,000 rpm for 10 min, filtered with a 0.22 μm filter to obtain the initial phage filtrate, and stored at 4°C. The bacteriophage was separated using the double-layer agar plate method (Mazzocco et al., 2009). The specific steps were as follows: 10 μl of bacteriophage filtrate was mixed with 100 μl of the Pa021 host bacterial solution. The mixture was incubated at room temperature for 8 min. Four millilitres of sterile LB semisolid culture medium (0.7%) were added at approximately 50°C, and the solution was immediately spread evenly onto sterile LB solid agar plates. The samples were allowed to dry and then placed in an incubator at 35°C. The next day, single plaques were selected and placed in 1 ml of sterile LB liquid medium, cultured at 37°C with shaking at 180 rpm for 40 min, and then centrifuged at 8,000 rpm for 3 min. The supernatant was obtained by filtration with a 0.22 μm sterile filter. Ten microlitres of phage lysate was mixed with 100 μl of the host bacteria Pa021 at the logarithmic growth phase, and the titre was determined by the double-layer agar plate method. This step was repeated 4 to 5 times until the size of the plaque on the double-layer agar plate was uniform, at which point a single bacteriophage was obtained (Matsuzaki et al., 2018). Pa021 was cultured to the early logarithmic stage, and 20 μl of the purified bacteriophages solution (approximately 10^9^ PFU/ml) was added to 2 ml of host bacteria solution, and the mixture was shaken and cultivated at 37°C and 180 rpm until the broth became clear. Then, the mixture was centrifuged at 8,000 rpm for 3 min and filtered using a 0.22 μm sterile filter to obtain the phage amplification liquid.

### 2.3 Determination of the optimal multiplicity of infection

The host bacterium Pa021 was cultured until it reached early logarithmic growth. The bacteriophage lysate was mixed with bacteria at different MOIs (10, 1, 0.1, 0.01, 0.001, and 0.0001), and the mixture was incubated for 4 h. One millilitre of mixed culture solution was removed from each tube, centrifuged at 8,000 rpm for 1 min, and filtered with a 0.22 μm sterile filter. The titre was determined by the double-layer agar plate method. The sample that produces the largest number of plaques is the optimal MOI of the bacteriophage (Nazir et al., 2022). The experiment was repeated three times.

### 2.4 Transmission electron microscopy observation

The purified and highly concentrated phage (approximately 10^12^ PFU/ml) was diluted 40 times with SM buffer and observed by transmission electron microscopy. The phage particles were fixed onto a copper grid using a carbon coating film and allowed to naturally adsorb for approximately 3 min. The samples were blotted dry with filter paper, negatively stained with 2% (w/v) phosphotungstic acid for 2 ~ 3 min, and then observed with an electron microscope after drying (FEI Tecnai G2 Spirit Bio TWIN Almeida et al., 2018).

### 2.5 Phage_Pae01 stability analysis

Phage lysates (approximately 10^10^ PFU/ml) were incubated at 4, 22, 37, 50, 60, or 70 for 1 h. The bacteriophage titre was measured under various temperature conditions. The pH of the sterile LB liquid medium was adjusted using HCl and NaOH to pH values of 1, 2, 3, 4, 5, 6, 7, 8, 9, 10, 11, and 12, respectively (Tsai et al., 2023). Ten microlitres of phage lysates (approximately 10^10^ PFU/ml) was added to 90 μl of liquid media with different pH values and cultured for 2 h at 37°C and 180 rpm (Akremi et al., 2022). The titre of the phage lysate was determined under different pH conditions, and the activity of the phage was evaluated. The experiment was repeated three times.

### 2.6 Phage_Pae01 host range assay

A total of 104 strains of *P. aeruginosa* were isolated from clinical specimens and environment and cultured into bacterial suspensions for host spectrum experiments. The bacterial suspension (100 μl) was mixed with sterile LB semisolid medium (4 ml) and then evenly spread onto a solid plate. Ten microlitres of the purified phage lysate was spotted on double-layer agar plates and incubated at 37°C overnight. The next day, we observed whether there was a clear cleavage area (Kutter, 2009). The experiment was repeated three times.

### 2.7 One-step growth curve of Phage_Pae01

The bacteriophage and host bacterium Pa021 were mixed according to the optimal multiplicity of infection (MOI, 0.01), incubated at room temperature for 8 min, centrifuged at 10000 rpm for 30 s to remove un-adsorbed phages. After washing twice with fresh LB medium, the pellet of infected cells was resuspended in 5 ml of LB medium and the culture was continuously incubated at 37°C. 100 μl sample was taken at each time point (0, 10, 20, 30, 60, 90, 120, and 150 min). Using double-layer agar plate method, we determined the free bacteriophage count at each time point. The burst size is the ratio of the final phage titre to the number of initially infected bacterial cells (Chen et al., 2020). The experiment was repeated three times.

### 2.8 Lysis kinetics of Phage_Pae01 *in vitro*

The host bacterium Pa021 was cultured to the logarithmic growth stage. In 96-well plates, 140 μl of sterile LB liquid medium and 30 μl of logarithmic host bacterial solution were added. Based on the different MOIs (10, 1, 0.1, and 0.01), 30 μl of phage lysate (10^9^ ~ 10^4^ PFU/ml) was added. In the control group, sterile LB liquid medium was used instead of bacteriophage, and each well was tested 3 times. The OD 600 was measured every 20 min to monitor bacterial growth using BIOSCREEN C° PRO (Lab Systems, Helsinki, Finland). The experiment was repeated three times (Horváth et al., 2020).

### 2.9 Extraction and sequencing of the Phage_Pae01 genome

To digest the DNA and RNA of *P. aeruginosa*, phage_Pae01 coarse particles were subjected to treatment with DNase I and RNase A at 37°C for 30 min. The mixture was then treated with EDTA (pH = 8.0) for 10 min at 65°C. A TIANamp Bacteria DNA Kit (Tiangen Biotech) was used to further prepare the DNA of the Phage_Pa01 genome. The genomic DNA of Phage_Pa01 was sequenced with an Illumina NovaSeq 6000 sequencer (Gu et al., 2019).

### 2.10 Genome analysis of Phage_Pae01

Phage_Pae01 was sequenced using Illumina NovaSeq 6,000 and PE150, and the sequencing results were assembled using SOAPdenovo-V2.04, SPAdes-3.12.0 and abyss 1.3.7 software. Finally, CISA (CISA1.3) software was used for integration, hole filling and optimization. The phage genome sequence was annotated using RAST online annotation software.^1^ Protein function was annotated using NCBI’s BLASTp tool (Altschul et al., 1990). Circular Genome Viewer (CGview; https://cgview.ca) was used to conduct phage genome-wide mapping. The virulence factors and antibiotic resistance genes were predicted using the virulence factor database (VFDB; http://www.mgc.ac.cn/VFs/) and comprehensive antibiotic research database (CARD; https://card.mcmaster.ca/). The whole-genome sequence was uploaded to BLASTn, and 3 similar phage sequences were selected and analysed with Phage_Pae01 via Easyfig (Rombouts et al., 2016). The phylogenetic tree was constructed based on the phage terminal large subunit using MEGA7.0 software (Kumar et al., 2016).

### 2.11 Effect of Phage_Pae01 and/or antibiotics on the biofilm formed

Previously reported methods were used, with some modifications, to determine the effect of phages and/or antibiotics on biofilm formation (Yang et al., 2022; Tsai et al., 2023). *P. aeruginosa* (1.0 × 10^7^ CFU/ml) was distributed in 100 μl aliquots to the wells of a 96-well microtiter plate and cultured at 35°C for 24 h. Thereafter, the LB medium was removed, and each well was gently washed three times with 100 μl of sterile phosphate-buffered saline (PBS). Then, 100 μl of Phage_Pae01 (3 × 10^9^ PFU/ml), Gentamicin (4 μg/ml, the MIC for *P. aeruginosa*), or Phage_Pae01 (3 × 10^9^ PFU/ml) + Gentamicin (4 μg/ml) was added. For the control group, 100 μl of LB was added. The samples were cultured at 35°C for 4, 8, or 24 h. At specific times, the medium was removed, and each well was washed three times with 100 μl of sterile PBS. After air drying at room temperature, 150 μl of 0.1% crystal violet was added to each well for 10 min. Then, the crystal violet was removed, and each well was gently washed three times with 150 μl of sterile PBS. Subsequently, 95% ethanol was added for elution, and the OD595 was measured using a microplate reader (Multiskan FC Akturk et al., 2023; Wang et al., 2023).

### 2.12 Effect of Phage_Pae01 on biofilm formation

Phage_Pae01 (50 μl) was added to the Pa021 culture (1.0 × 10^7^ CFU/ml) at an MOI of 100. LB medium without phage was used as a control. A total of 100 μl of the culture was added to 96-well plates. Three identical plates were prepared together and cultured at 35°C for 4, 8, or 24 h. Then, the OD595 was measured as previously described (Yang et al., 2022).

### 2.13 Scanning electron microscopy (SEM) observation

With some modifications, SEM analysis was conducted using a previously described method (Mulani et al., 2022). Specifically, 1 ml of *P. aeruginosa* Pa021 (1.0 × 10^7^ CFU/ml) was added to a 12-well plate (Corning Corp., USA) that contained 14 mm cell slides at the bottom. The plate was then incubated at 35°C for 24 h. The slides containing the cells were removed and washed three times with sterile PBS. Next, the cell slides were placed into new wells. Then, 1 ml of Phage_Pae01 (3 × 10^9^ PFU/ml), Gentamicin (4 μg/ml, the MIC for *P. aeruginosa*), or Phage_Pae01 (3 × 10^9^ PFU/ml) + Gentamicin (4 μg/ml) was added. For the control group, 1 ml of LB was added. The samples were cultured at 35°C for 24 h. The liquid culture medium was removed, and the cell slides were washed with PBS. Each slide was immobilized with 3% glutaraldehyde at 4°C overnight, and then washed 4 times with PB solution for 10 min. OSmic acid was added for 1 h. The samples were rinsed three times with deionized water, followed by double dehydration with a gradient ethanol series (50, 70, 90, and 100%). Then, the cell slides were placed on filter paper, dried at the zero point for 2 h, and coated with gold. Finally, a scanning electron microscope (JEOL JSM-7900F) was used to observe the size and morphology of the biofilm (Mulani et al., 2022; Vashisth et al., 2023).

### 2.14 Statistical analysis

We used GraphPad Prism 9.4.1 software for the data analysis. The results of the assays were compared using one-way analysis of variance (ANOVA) by applying the Tukey’s multiple comparisons test. The unpaired *t* test for differences in variance was used. Differences were considered statistically significant at *p* < 0.05.

## 3 Results

### 3.1 Plaque and phage morphology of Phage_Pae01

**Figure 1:**
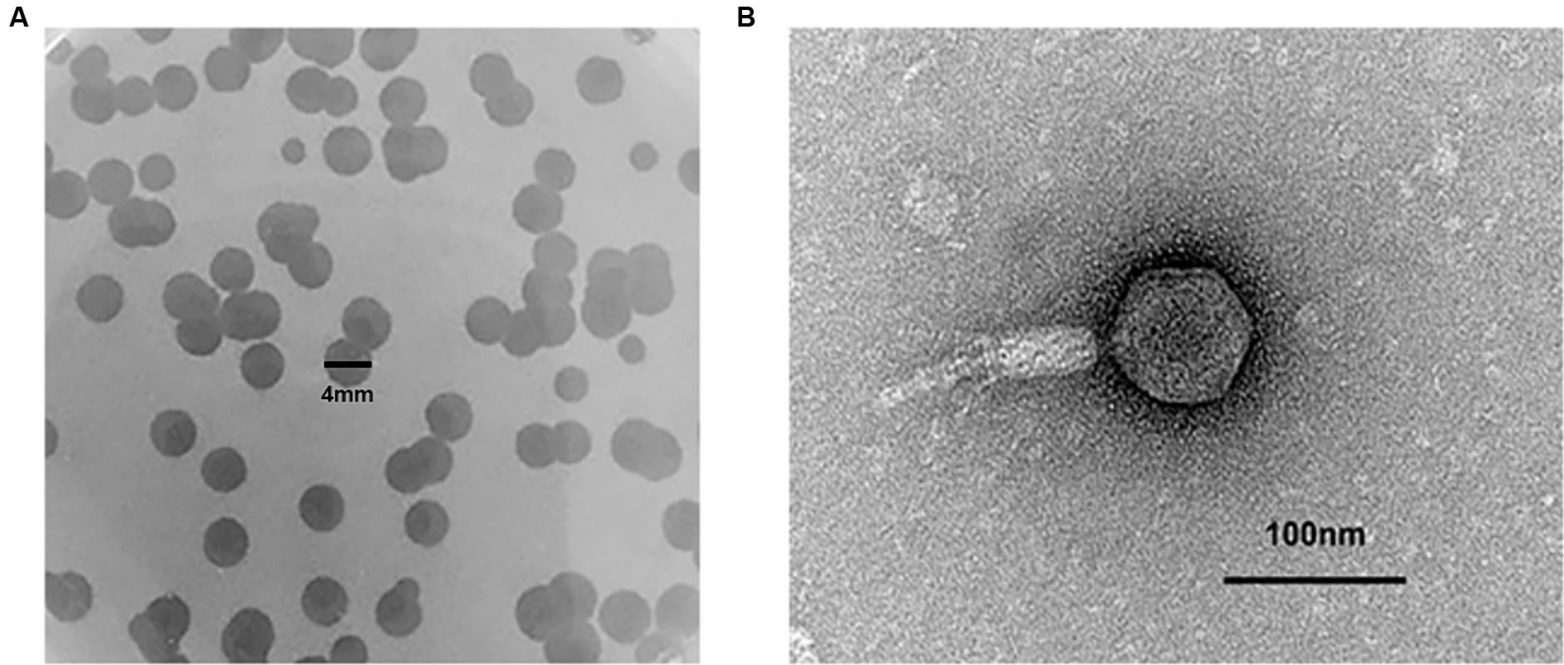
The plaque and phage morphology of Phage_Pae01. **(A)** Plaques of Phage_Pae01 to the host strain Pa021, were clear with an expanding halo. **(B)** Morphology of Phage_Pae01 by transmission electron microscopy. The scale bar represents 100 nm.

*P. aeruginosa* Pa021 isolated from the sewage treatment centre of Jiangsu Women and Children Health Hospital was used as a host bacterium. The untreated sewage was used for phage isolation. To increase the likelihood of acquiring target phages, after removing sediment, the sewage was co-cultured with host bacteria. In the process of culture, fresh LB was added as a nutrient to enrich phages in the sewage. During the plaque experiment, a significant amount of transparent plaque was observed. Bacteriophages are present wherever bacteria are present. A complete and transparent plaque was randomly selected for further purification and amplification. A single phage, Phage_Pae01, was isolated when uniform plaques were obtained. Phage_Pae01 formed clear plaques with a diameter of 3 to 4 mm on the plate of the host bacteria (Figure 1A). Transmission electron microscopy revealed that the phage possessed an icosahedral head of approximately 80 nm and a long tail about 110 nm, indicating that it belonged to the *Myoviridae* family of the order *Caudovirales* (Figure 1B; Adriaenssens and Brister, 2017).

### 3.2 MOI, one-step growth curve and lysis kinetics of Phage_Pae01

**Figure 2:**
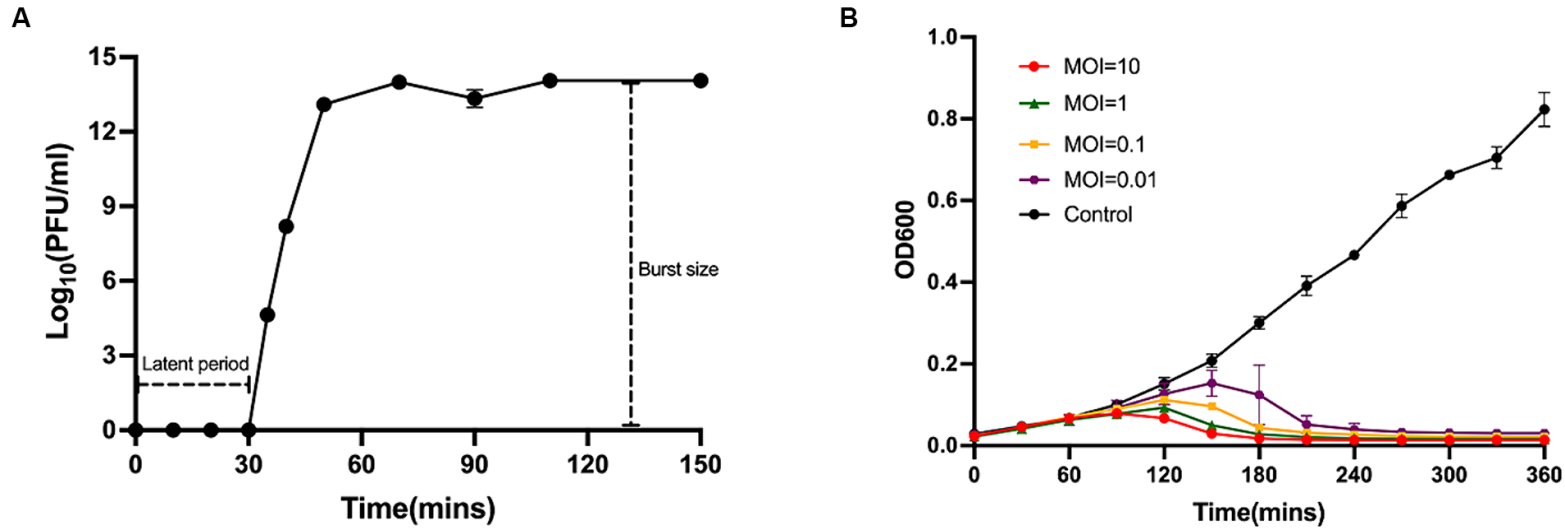
One-step growth curve and lysis kinetics of Phage_Pae01. **(A)** One-step growth curve of Phage_Pae01 on *P. aeruginosa* strain Pa021. **(B)** Killing curves of *P. aeruginosa* strain Pa021 by Phage_Pae01 at various MOIs (10, 1, 0.1, and 0.01).

In the MOI assay, we observed that the highest titre of phage lysate was obtained when the ratio of phage to bacteria was 0.01. Therefore, the optimal MOI for Phage_Pae01 was 0.01. The one-step growth curve demonstrated that the latency period of Phage_Pae01 was 30 min. Afterwards, the number of bacteriophage offspring in the solution gradually increased. After 60 min, the number of bacteriophages in the system began to stabilize, reaching 10^14^ PFU/ml (Figure 2A). Furthermore, the burst size of Phage_Pae01 is about 10^6^ average progeny per infected cell. Different MOIs were used to monitor the bacteriolytic activity of the phage, with Pa021 serving as the host (Figure 2B). At approximately 120 min, the growth of bacteria was gradually inhibited compared with that in the control group. In the control group without phages, the concentration of bacteria gradually increased. When the MOI was 0.01, the growth of the bacteria was still inhibited, and the bactericidal effect persisted for up to 9 h.

### 3.3 Host range of Phage_Pae01

The lytic ability of Phage_Pae01 was determined by spot and plaque assays. We found that Phage_Pae01 could effectively lyse 87 out of the 104 strains of *P. aeruginosa* (83.6%), indicating a large host range. When Phage_Pae01 formed a clear plaque in the designated area on the bacterial plate, it indicates that the phage has strong lytic ability to the tested bacteria. The results showed that phage Phage_Pae01 showed strong cleavage ability to 82 of the 87 sensitive strains, and the remaining 5 strains showed turbid plaque. These tested strains included carbapenem-resistant, multidrug-resistant, and standard *P. aeruginosa* strains (Table 1).

**Table 1:** Host range of Phage_Pae01 against *P. aeruginosa.*

*P. aeruginosa* subtype	Host range
Sensitive	54/65(83.1%)
CRPA	28/31(90.3%)
MDRPA	33/39 (84.6%)
Total	87/104 (83.6%)

### 3.4 Phage_Pae01 temperature and pH stability

**Figure 3:**
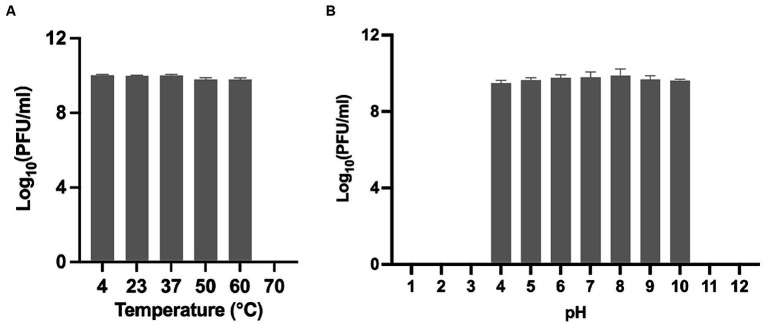
Biological properties of Phage_Pae01. **(A)** Thermal stability of Phage_Pae01. Phages were incubated for 1 h at different temperatures. **(B)** pH stability of Phage_Pae01. Phages were incubated for 2 h at different pHs.

To obtain a comprehensive understanding of Phage_Pae01 and lay a foundation for future phage therapy, we conducted further investigations of the stability of this phage under various environmental conditions. After incubating Phage_Pae01 at different temperatures for a certain period of time, we found that Phage_Pae01 could still maintain high activity at temperatures up to 60°C (Figure 3A). Under acidic conditions at pH 4 and alkaline conditions at pH 10, Phage_Pae01 activity decreased only slightly (Figure 3B). The new Phage_Pae01 is a good option for phage therapy.

### 3.5 Genome analysis of Phage_Pae01

**Figure 4:**
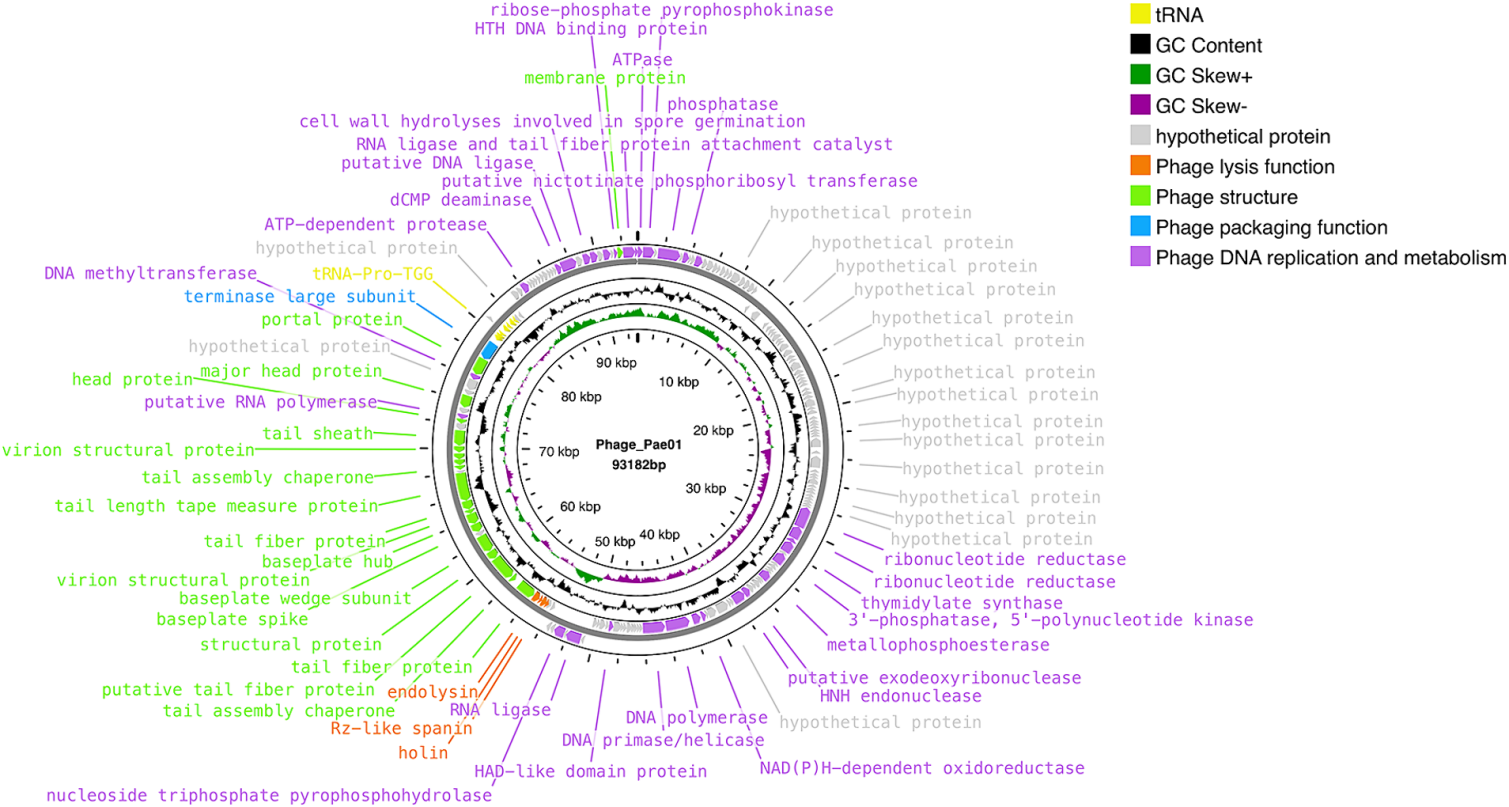
Whole-genome map of Phage_Pae01.

**Figure 5:**
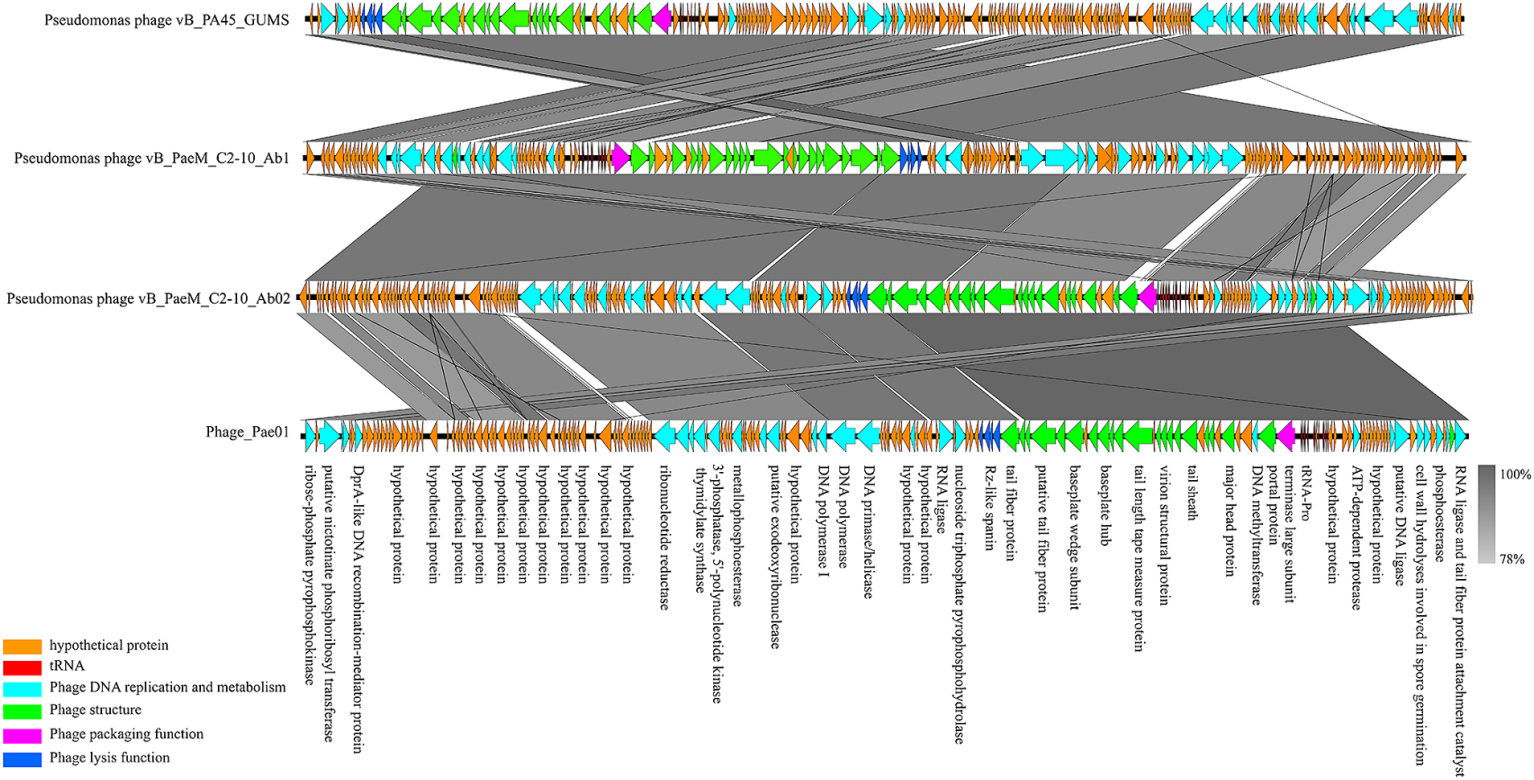
Comparative genome analysis of the *P. aeruginosa* phages Phage_Pae01, vB_PA45_GUMS, vB_PaeM_C2-10_Ab1 and vB_PaeM_C2-10_Ab02. The direction of transcription is shown by arrows next to the predicted ORFs. According to the key provided at the bottom left of the graphic, the arrows are coloured according to their functions. The corresponding ORFs are presented with functional annotations.

**Figure 6:**
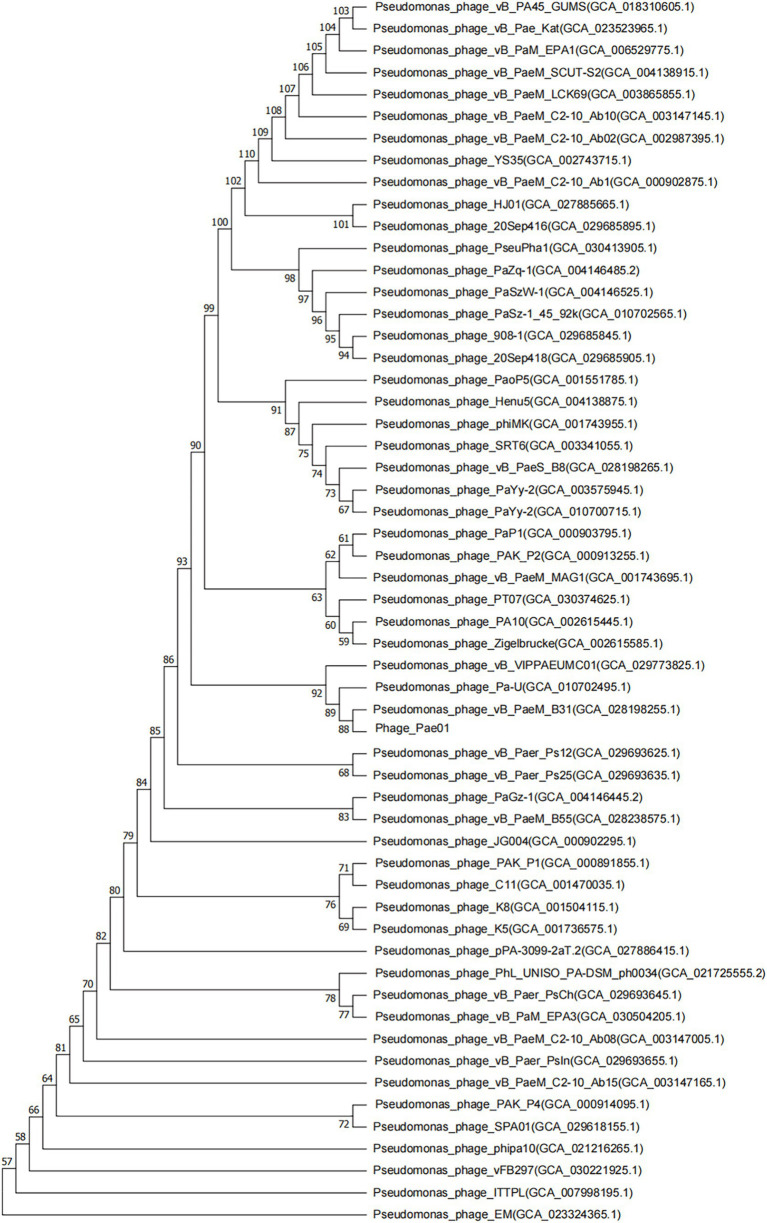
Phylogenetic tree based on the phage terminal large subunit comparisons of selected phages. Amino acid sequence comparison was compared using the ClustalW program, and the phylogenetic trees were generated using the neighbor-joining method with 1,000 bootstrap replications. The numbers at the nodes represent the percent bootstrap values. The numbers in parentheses represent the GenBank accession number.

Phage_Pae01 is a double-stranded DNA phage with a total genome size of 93,182 bp and a GC content of 49.4%. CARD database annotation results showed no antibiotic resistance genes. Virulence genes were not detected by VFDB database comparison, indicating that Phage_Pae01 is safe. The functional genes of Phage_Pae01, which encode a variety of phage structural protein genes, are diverse. Additionally, the genome of Phage_Pae01 contains 176 open reading frames (ORFs). NCBI BLASTp was used to annotate phage functional genes, and the results are shown in Supplementary Table S3. Among them, 119 ORFs were hypothetical proteins or proteins of unknown function, and the remaining 57 ORFs were functional coding sequences (CDSs). The genome annotation map is shown in Figure 4. Among the ORFs, proteins associated with phage DNA replication and metabolism, such as ORF166 (putative DNA ligase), which encodes a phage-associated DNA ligase, were identified. This ligase serves as a transcriptional switch that prevents the transcription of early phage genes and enables the transcription of mid-to-late genes. Structural proteins such as ORF122 and 132 are tail fibre proteins that have contraction and motor functions. This gene cluster plays an important role in bacteriophage infection and the adsorption of host bacteria. ORF121 encodes endolysin, which hydrolyses the peptidoglycan of the cell wall. ORF120 is a holin. When holin forms micron-sized pores in the bacterial inner membrane, it initiates cleavage and releases active endolysin into the periplasmic space to degrade peptidoglycans. ORF118 and 119 encode Rz-like spanins, which can destroy the outer membrane of host cells and participate in cell lysis (Liu et al., 2022). Packaging proteins such as ORF149 (terminal enzyme large subunit) have endonuclease activity and can precisely cut the viral genome to initiate and end packaging reactions. Phage_Pae01 was relatively similar to Pseudomonas phages vB_PaeM_C2-10_Ab02 (96% coverage, 97.47% identity, accession number NC_042113.1), vB_PaeM_C2-10_Ab1 (95% coverage, 97.19% identity, accession number NC_019918.1) and vB_PA45_GUMS (94% coverage, 97.2% identity, accession number NC_073619.1), indicating that these phages may belong to the same genus. Easyfig analysis further showed that Phage_Pae01 was highly homologous to the aforementioned phages (Figure 5). The large phage terminase subunit is highly conserved; thus, the large terminase subunit of Phage_Pae01 was investigated for phylogenetic tree construction (Figure 6).

### 3.6 Effect of Phage_Pae01 and/or antibiotics on the biofilm formed

**Figure 7:**
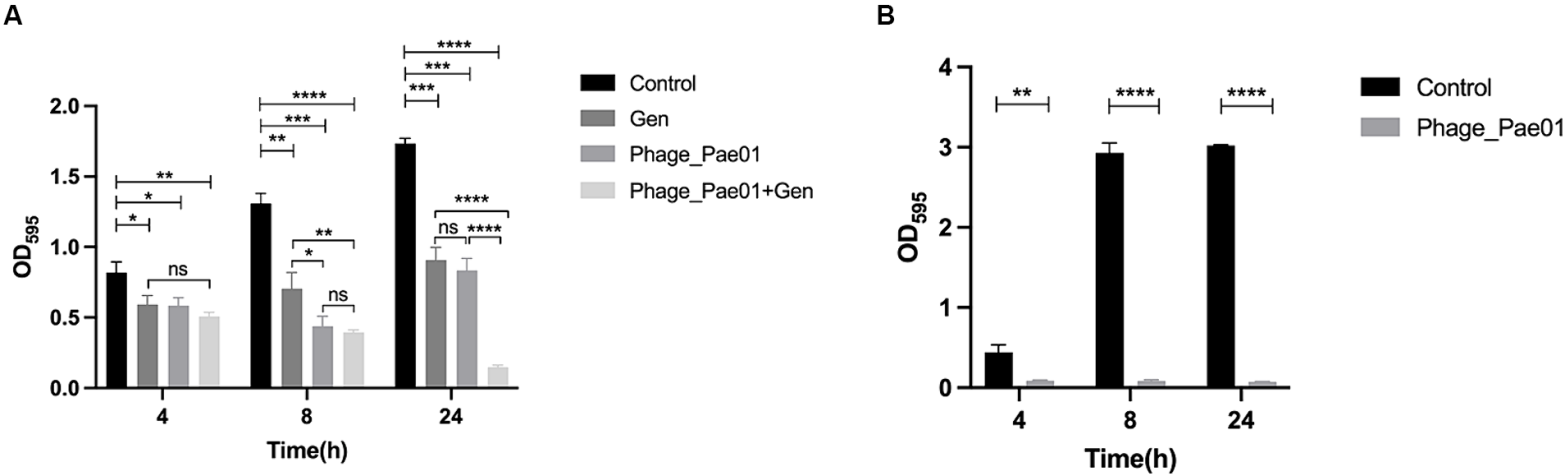
Effects of the phage on biofilms. **(A)** Effects of Phage_Pae01 and/or antibiotics on preformed biofilms. The effects of Phage_Pae01 and/or antibiotics on the preformed biofilm at 4, 8, and 24 h are shown. *p < 0.05, **p < 0.01, ***p < 0.0005, ****p < 0.0001. **(B)** Effects of phage coincubation on biofilm formation. The effects of Phage_Pae01 on biofilm formation after 4, 8, and 24 h of coincubation are shown. **p < 0.01, ****p < 0.0001. **(B)** Effects of phage co-incubation on biofilm formation. The effects of Phage_Pae01 on biofilm formation after 4, 8, and 24 hours of co-incubation are shown. **p < 0.01, ****p < 0.0001.

**Figure 8:**
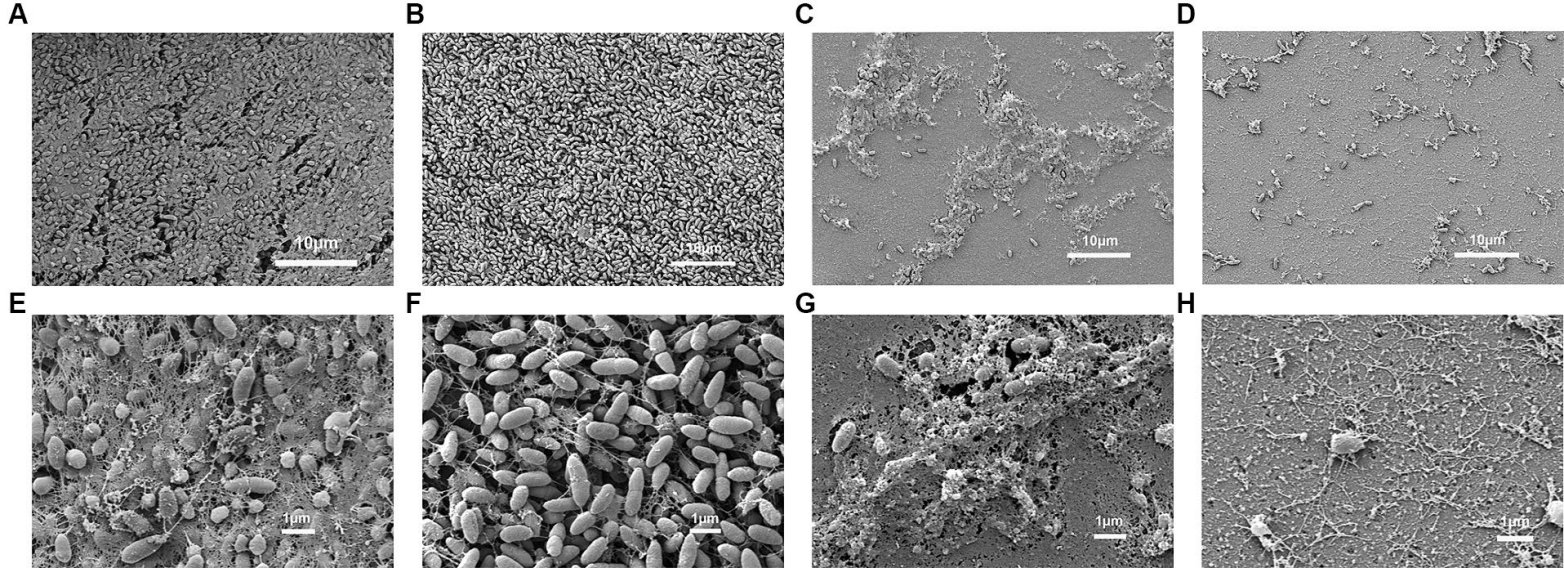
Scanning electron microscopy images of *P. aeruginosa* Pa021 treated with Phage_Pae01 and/or antibiotics: **(A, E)** *P. aeruginosa* Pa021; **(B, F)** *P. aeruginosa* Pa021 + Gentamicin; **(C, G)** *P. aeruginosa* Pa021 + Phage_Pae01; and **(D, H)** *P. aeruginosa* Pa021 + Gentamicin + Phage_Pae01.

As shown in Figure 7A, after the formation of *P. aeruginosa* biofilms for 24 h, phage treatment alone for 4 h had a slight destructive effect on the biofilm compared with that of the control group. Phage treatment alone for 8 or 24 h significantly disrupted the bacterial biofilm. The effect of gentamicin alone on the biofilm was not as significant as that of Phage_Pae01. However, when the biofilm was treated with Phage_Pae01 in combination with gentamicin, the disruption of the bacterial biofilm was greater than that of the phage or antibiotic alone. This result indicates that there is a remarkable synergistic effect between bacteriophages and antibiotics in the elimination of bacterial biofilms. Figures 8A,E show the *P. aeruginosa* biofilms in the control group at different magnifications. Figures 8B,F show the biofilm after treatment with gentamicin at the MIC for 24 h. Compared with those in the control group, the biofilms were slightly sparser, but there were still more free bacteria and surface bacteria. Figures 8C,G show the biofilm after treatment with Phage_Pae01 for 24 h. Compared with those in the control group and the antibiotic treatment group, the dense biofilm structure was destroyed, and the free bacteria, surface bacteria and inner bacteria was significantly reduced. Phages can penetrate bacterial biofilms and infect inner bacteria, resulting in lysis. Figures 8D,H demonstrate the biofilm status after 24 h of combined treatment with Phage_Pae01 and gentamicin. There was a significant reduction in free bacteria, surface bacteria, and inner bacteria compared to that in the control group. Additionally, the dense biofilm structure was completely destroyed.

### 3.7 Effect of Phage_Pae01 on biofilm formation

As shown in Figure 7B, when Phage_Pae01 and its host bacterium Pa021 were co-cultured at an MOI of 100 for 4, 8, or 24 h, the formation of bacterial biofilms was inhibited compared to that in the control group without phage. Thus, the phage has the ability to effectively inhibit the formation of bacterial biofilms on object surfaces, making it a promising agent for disinfection.

## 4 Discussion

*P. aeruginosa* is one of the main pathogens that causes nosocomial infection (Singh et al., 2021; Akturk et al., 2023). The resistance of *P. aeruginosa* to antibiotic therapy through intrinsic resistance and acquired resistance makes the clinical treatment of *P. aeruginosa* infection particularly difficult. Furthermore, *P. aeruginosa* is a gram-negative bacterium that is likely to form bacterial biofilms (Sauer et al., 2022). The production of biofilms increases resistance to antibiotics, disinfectants, and the immune system, thereby making the control of *P. aeruginosa* even more challenging. Therefore, there is an urgent need to discover alternative treatments aside from antibiotics. Bacteriophages are viruses that infect and kill bacteria. Due to the critical situation of drug-resistant bacteria, bacteriophages are once again being utilized for the treatment of clinical infectious diseases.

In this study, *P. aeruginosa* Pa021 was isolated from sewage as a host bacterium, and the lytic phage Phage_Pae01 was isolated from the sewage of Jiangsu Women and Children Health Hospital. Transmission electron microscopy revealed that the phage possessed an icosahedral head of approximately 80 nm and a long tail about 100 nm, indicating its classification within the *Myoviridae* family of the order *Caudovirales*. The latency period of the phage was 30 min, the lysis period was 60 min, and the burst size was about 10^6^. Although the latent period was longer, the burst size of Phage_Pae01 was significantly greater than those of most previously reported *P. aeruginosa* phages (Wannasrichan et al., 2022; Abdelghafar et al., 2023; Teklemariam et al., 2023). Thus, a longer incubation period leads to the production of a greater number of progeny phages. *In vitro* cleavage experiment, compared with the control group, the growth of host bacteria was inhibited under different MOI conditions, showing strong cleavage ability. Phage Phage_Pae01 has the ability to infect 83.6% (87/104) of the *P. aeruginosa* strains tested across various hospitals. Tsai et al. isolated the phage phiPA1-3 of *P. aeruginosa*, which exhibited a lysis rate of 20% (Tsai et al., 2023). Fei et al. isolated a broad-spectrum *P. aeruginosa* phage HZ2201 with a cleavage rate of 78.38% (Fei et al., 2023). It shows that Phage_Pae01 has a wide cleavage spectrum, overcomes the problem of narrow host spectrum in phage therapy, and can be used as a candidate for phage therapy. Testing the stability of the phage under different conditions can help better understand the basic conditions for the phage to function. In order to gain a more comprehensive understanding of phage Phage_Pae01, we further explored its activity under different temperature and pH conditions. After incubation at different temperatures for 1 h, phage Phage_Pae01 still maintained high activity at a temperature of up to 60°C, while phage phiPA1-3 had a survival rate of about 40% at a temperature of 50°C. The activity of phage HZ2201 also decreased with the increase of temperature (Fei et al., 2023; Tsai et al., 2023). It can be seen that phage Phage_Pae01 can still maintain a high activity state at a higher temperature. In addition, Phage_Pae01 maintained good titer and activity over a wide pH range (4 ~ 10), similar to most phages that have been reported (Akremi et al., 2022; Wannasrichan et al., 2022).

Genome analysis showed that phage Phage_Pae01 had a total genome size of 93,182 bp and a GC content of 49.4%. According to the annotation results, the functional protein is divided into four modules: phage DNA replication and metabolic protein, structural protein, lysis protein and packaging protein. For dsDNA phages, the cleavage system involves two or more proteins, and typically these proteins include holin and endolysin (Chamblee et al., 2022). A protein associated with host cleavage, ORF121 encodes an endolysin, which hydrolyses the peptidoglycan of the cell wall; ORF120 is a holin. Holin is responsible for controlling the timing of cleavage, and all dsDNA phages utilize holin to affect bacterial cleavage. When holin forms micron-sized pores in the intima, it initiates cleavage and releases active endolysin into the periplasmic space to degrade peptidoglycans. The formation of these pores causes the host’s metabolism to stop, as the proton power that fuels ATP synthesis is destroyed (Wang et al., 2003). ORF118 and 119 encode Rz-like spanin, which can destroy the outer membrane of host cells and play a role in cell lysis. Once the final cellular barrier is breached, the pressure from swelling cells causes the cytoplasmic contents, including progeny phages, to be forcefully expelled into the surrounding medium.

Wannasrichan W. showed that the combination of bacteriophages and antibiotics can effectively inhibit the growth and biofilm formation of *P. aeruginosa* (Wannasrichan et al., 2022). In this study, the aminoglycoside antibiotic gentamicin was combined with Phage_Pae01 to treat *P. aeruginosa* biofilms. We discovered that Phage_Pae01 alone led to more significant clearance of biofilms than gentamicin alone. The combined use of the phage and an aminoglycoside antibiotic had a synergistic effect on biofilm clearance, which was greater than that of either drug alone. The biofilm inside the human body is able to resist host immunity and its sensitivity to antibiotics is much lower than that of the same species of free bacteria. EPS (extracellular polymer), which makes up the biofilm, can inhibit the penetration of antibiotics and greatly reduce the effective dose of antibiotics entering the biofilm. The resistance of the biofilm to antibiotics also allows the bacteria time to adapt and develop resistance to antibiotics. As the natural enemy of bacteria, bacteriophages can enter the biofilm and play a role in many ways to break the first physical barrier formed by the extracellular matrix of the biofilm (Chang et al., 2022). Phages encode a variety of lytic proteins, such as virion-associated peptidoglycan hydrolases (VAPGHs), holin, endolysin, etc., which can naturally penetrate the extracellular matrix of biofilm and enhance its killing effect on biofilm (Rahman et al., 2021). In the whole genome of phage Phage_Pae01, annotation results showed that ORF120 encodes holin, which is mainly responsible for destroying cell membrane components. ORF121 encodes endolysin, which destroys bacterial cell walls and acts in the peptidoglycan layer (Chandran et al., 2022). This allows the bacteriophages to quickly penetrate the biofilm and lyse the bacterial cells.

Phages can use the water channel structure of biofilms to break the diffusion limit of biofilms. The diffusion limitation of the biofilm will result in the decrease of the antibiotic concentration in the inner biofilm layer, and the phage will increase the amount through active replication during the diffusion process (Figueiredo et al., 2021). Once the water channel is opened, the phage can assist the antibiotic to enter the interior of the biofilm. Phages, unlike antibiotics, have the ability to infect persistent cells. Although bacteria are sensitive to antibiotics, high concentrations of antibiotics do not kill bacterial persistent cells (Rather et al., 2021). When a single dose of phage is used to clear a biofilm, the proliferation of phage-insensitive mutants may result if the phage does not rapidly kill a sufficient number of bacteria (Simmons et al., 2020; Pinto et al., 2021). At the later stage of the combined treatment experiment, phage and gentamicin had synergistic effects. Bacteriophages are responsible for eliminating antibiotic-resistant bacteria, while antibiotics are responsible for eliminating bacteriophage-resistant bacteria (Chaudhry et al., 2017; Simmons et al., 2020). Consequently, there was a noteworthy decrease in the quantity of biofilm bacteria after combined treatment compared to single-dose treatments. de Cassia Oliveira et al. reported that after 24 h of *P. aeruginosa* and phage co-culture, compared with the control treatment, all phages effectively reduced the growth rate of biofilms in the early stages of biofilm formation (Oliveira et al., 2021). However, after 24 h of co-culture, there was no significant difference in biofilm formation between the control group and the phage-treated group. In the co-culture experiment with Phage_Pae01 and the host bacterium Pa021, compared with that in the control group, the growth of *P. aeruginosa* biofilms was still inhibited 24 h after co-culture. This result indicated that Phage_Pae01 could effectively and sustainably inhibit the formation of bacterial biofilms and was a good candidate for biofilm prevention and control.

In conclusion, Phage_Pae01 has a wide host spectrum and strong lytic ability. Moreover, Phage_Pae01 can prevent and control the formation of bacterial biofilms, as it effectively eliminated 24-h bacterial biofilms. A synergistic effect in clearing bacterial biofilms is observed when bacteriophages are combined with aminoglycoside antibiotics. These properties indicate that Phage_Pae01 is an effective therapeutic agent.

## Data availability statement

The complete phage sequence has been submitted to GenBank under the accession number OR858750.

## Ethics statement

The studies involving humans were approved by the Ethics Committee of Jiangsu Province Hospital (IRB approved number: 2024-SR-075). The present study was a study focusing on bacteria isolates and did not contain any sensitive personal information. Therefore, informed consent was not required. The studies were conducted in accordance with the local legislation and institutional requirements. Written informed consent for participation was not required from the participants or the participants’ legal guardians/next of kin in accordance with the national legislation and institutional requirements.

## Author contributions

ZS: Conceptualization, Data curation, Formal analysis, Investigation, Methodology, Software, Writing – original draft, Writing – review & editing. XH: Data curation, Investigation, Methodology, Writing – review & editing. ZL: Data curation, Investigation, Methodology, Writing – review & editing. MZ: Methodology, Resources, Writing – review & editing. JZ: Methodology, Resources, Writing – review & editing. ZZ: Methodology, Resources, Writing – review & editing. SQ: Project administration, Resources, Writing – review & editing. GL: Conceptualization, Funding acquisition, Project administration, Resources, Supervision, Validation, Writing – review & editing.

## Footnotes

^1^ https://rast.nmpdr.org/
