# Supplementary material for: Characterization of the novel broad-spectrum lytic phage Phage_Pae01 and its antibiofilm efficacy against Pseudomonas aeruginosa
Source: Front Microbiol. 2024 Jul 17;15:1386830. doi: 10.3389/fmicb.2024.1386830 (PMC11292732; doi:10.3389/fmicb.2024.1386830)
Supplement: Supplementary file 1 [file Table_1.DOCX]

Supplementary Material

# Supplementary Tables

## Supplementary Table S1

**The source and antibiotic resistance information of all *P. aeruginosa* strains.**

| Strain No | Drug resistance | Strain | Source | Plaque  formation |
| --- | --- | --- | --- | --- |
| Pa001 | S | Pseudomonas aeruginosa | Wound secretion | +++ |
| Pa002 | S | Pseudomonas aeruginosa | Pus | +++ |
| Pa003 | MDRP | Pseudomonas aeruginosa | Sputum | +++ |
| Pa004 | S | Pseudomonas aeruginosa | Duct | +++ |
| Pa005 | CRPA | Pseudomonas aeruginosa | Wound secretion | +++ |
| Pa006 | CRPA | Pseudomonas aeruginosa | Alveolar lavage fluid | +++ |
| Pa007 | CRPA | Pseudomonas aeruginosa | Sputum | +++ |
| Pa008 | CRPA | Pseudomonas aeruginosa | Sputum | - |
| Pa009 | MDRP | Pseudomonas aeruginosa | Sputum | - |
| Pa010 | MDRP | Pseudomonas aeruginosa | Sputum | +++ |
| Pa011 | S | Pseudomonas aeruginosa | Sputum | +++ |
| Pa012 | CRPA | Pseudomonas aeruginosa | Sputum | +++ |
| Pa013 | S | Pseudomonas aeruginosa | Pus | +++ |
| Pa014 | S | Pseudomonas aeruginosa | Ascites | +++ |
| Pa015 | CRPA | Pseudomonas aeruginosa | Sputum | +++ |
| Pa016 | CRPA | Pseudomonas aeruginosa | Sputum | +++ |
| Pa017 | CRPA | Pseudomonas aeruginosa | Sputum | +++ |
| Pa018 | CRPA | Pseudomonas aeruginosa | Sputum | +++ |
| Pa019 | CRPA | Pseudomonas aeruginosa | Sputum | - |
| Pa020 | CRPA | Pseudomonas aeruginosa | Alveolar lavage fluid | +++ |
| Pa021 | S | Pseudomonas aeruginosa | Sewage | +++ |
| Pa022 | S | Pseudomonas aeruginosa | Laboratory | +++ |
| Pa023 | S | Pseudomonas aeruginosa | Laboratory | +++ |
| Pa024 | S | Pseudomonas aeruginosa | Laboratory | +++ |
| Pa025 | S（ATCC27853） | Pseudomonas aeruginosa | Laboratory | +++ |
| Pa026 | S | Pseudomonas aeruginosa | Soil | - |
| Pa027 | S | Pseudomonas aeruginosa | Oropharyngeal secretion | +++ |
| Pa028 | S | Pseudomonas aeruginosa | Sputum | - |
| Pa029 | CRPA | Pseudomonas aeruginosa | Sputum | +++ |
| Pa030 | CRPA | Pseudomonas aeruginosa | Sputum | +++ |
| Pa031 | S | Pseudomonas aeruginosa | Sputum | +++ |
| Pa032 | CRPA | Pseudomonas aeruginosa | Sputum | +++ |
| Pa033 | S | Pseudomonas aeruginosa | Skin secretion | +++ |
| Pa034 | S | Pseudomonas aeruginosa | Sputum | +++ |
| Pa035 | S | Pseudomonas aeruginosa | Sputum | +++ |
| Pa036 | S | Pseudomonas aeruginosa | Sputum | + |
| Pa037 | S | Pseudomonas aeruginosa | Sputum | - |
| Pa038 | S | Pseudomonas aeruginosa | Sputum | +++ |
| Pa039 | CRPA | Pseudomonas aeruginosa | Sputum | +++ |
| Pa040 | S | Pseudomonas aeruginosa | Sputum | - |
| Pa041 | S | Pseudomonas aeruginosa | Sputum | +++ |
| Pa042 | CRPA | Pseudomonas aeruginosa | Sputum | - |
| Pa043 | CRPA | Pseudomonas aeruginosa | Ascites | + |
| Pa044 | S | Pseudomonas aeruginosa | Skin secretion | +++ |
| Pa045 | CRPA | Pseudomonas aeruginosa | Sputum | +++ |
| Pa046 | S | Pseudomonas aeruginosa | Sputum | +++ |
| Pa047 | S | Pseudomonas aeruginosa | Alveolar lavage fluid | +++ |
| Pa048 | S | Pseudomonas aeruginosa | Sputum | +++ |
| Pa049 | S | Pseudomonas aeruginosa | Sputum | +++ |
| Pa050 | CRPA | Pseudomonas aeruginosa | Sputum | +++ |
| Pa051 | S | Pseudomonas aeruginosa | Sputum | +++ |
| Pa052 | S | Pseudomonas aeruginosa | Sputum | +++ |
| Pa053 | S | Pseudomonas aeruginosa | Sputum | +++ |
| Pa054 | S | Pseudomonas aeruginosa | Sputum | - |
| Pa055 | S | Pseudomonas aeruginosa | Sputum | +++ |
| Pa056 | S | Pseudomonas aeruginosa | Sputum | +++ |
| Pa057 | CRPA | Pseudomonas aeruginosa | Sputum | +++ |
| Pa058 | S | Pseudomonas aeruginosa | Sputum | +++ |
| Pa059 | S | Pseudomonas aeruginosa | Alveolar lavage fluid | +++ |
| Pa060 | S | Pseudomonas aeruginosa | Sputum | +++ |
| Pa061 | S | Pseudomonas aeruginosa | Sputum | +++ |
| Pa062 | S | Pseudomonas aeruginosa | Sputum | +++ |
| Pa063 | S | Pseudomonas aeruginosa | Sputum | +++ |
| Pa064 | S | Pseudomonas aeruginosa | Sputum | +++ |
| Pa065 | CRPA | Pseudomonas aeruginosa | Sputum | +++ |
| Pa066 | CRPA | Pseudomonas aeruginosa | Sputum | +++ |
| Pa067 | MDRP | Pseudomonas aeruginosa | Sputum | - |
| Pa068 | S | Pseudomonas aeruginosa | Sputum | +++ |
| Pa069 | MDRP | Pseudomonas aeruginosa | Sputum | - |
| Pa070 | S | Pseudomonas aeruginosa | Sputum | +++ |
| Pa071 | CRPA | Pseudomonas aeruginosa | Sputum | +++ |
| Pa072 | S | Pseudomonas aeruginosa | Alveolar lavage fluid | +++ |
| Pa073 | S | Pseudomonas aeruginosa | Urine | +++ |
| Pa074 | S | Pseudomonas aeruginosa | Sputum | +++ |
| Pa075 | CRPA | Pseudomonas aeruginosa | Sputum | +++ |
| Pa076 | S | Pseudomonas aeruginosa | Sputum | +++ |
| Pa077 | S | Pseudomonas aeruginosa | Sputum | +++ |
| Pa078 | S | Pseudomonas aeruginosa | Urine | + |
| Pa079 | CRPA | Pseudomonas aeruginosa | Sputum | + |
| Pa080 | S | Pseudomonas aeruginosa | Alveolar lavage fluid | +++ |
| Pa081 | S | Pseudomonas aeruginosa | Sputum | +++ |
| Pa082 | S | Pseudomonas aeruginosa | Sputum | +++ |
| Pa083 | CRPA | Pseudomonas aeruginosa | Wound secretion | +++ |
| Pa084 | CRPA | Pseudomonas aeruginosa | Sputum | +++ |
| Pa085 | CRPA | Pseudomonas aeruginosa | Sputum | +++ |
| Pa086 | CRPA | Pseudomonas aeruginosa | Sputum | +++ |
| Pa087 | CRPA | Pseudomonas aeruginosa | Urine | +++ |
| Pa088 | CRPA | Pseudomonas aeruginosa | Sputum | +++ |
| Pa089 | S | Pseudomonas aeruginosa | Sputum | - |
| Pa090 | S | Pseudomonas aeruginosa | Sputum | +++ |
| Pa091 | S | Pseudomonas aeruginosa | Sputum | +++ |
| Pa092 | S | Pseudomonas aeruginosa | Skin secretion | + |
| Pa093 | MDRP | Pseudomonas aeruginosa | Sputum | +++ |
| Pa094 | S | Pseudomonas aeruginosa | Sputum | - |
| Pa095 | S | Pseudomonas aeruginosa | Sputum | +++ |
| Pa096 | S | Pseudomonas aeruginosa | Sputum | - |
| Pa097 | S | Pseudomonas aeruginosa | Sputum | +++ |
| Pa098 | S | Pseudomonas aeruginosa | Sputum | - |
| Pa099 | MDRP | Pseudomonas aeruginosa | Alveolar lavage fluid | +++ |
| Pa100 | S | Pseudomonas aeruginosa | Sputum | - |
| Pa101 | S | Pseudomonas aeruginosa | Sputum | +++ |
| Pa102 | S | Pseudomonas aeruginosa | Sputum | - |
| Pa103 | MDRP | Pseudomonas aeruginosa | Sputum | +++ |
| Pa104 | S | Pseudomonas aeruginosa | Sputum | +++ |

*Clear plaque*: “+++”. *Turbid plaque*. “+”. *No plaque*: “-”.

## Supplementary Table S2

## Antibiotic resistance profiling of *P. aeruginosa* Pa021.

| Antimicrobial Agent | Pa021 | Antibiotic sensitivity |
| --- | --- | --- |
| Piperacillin | 8 | S |
| Piperacillin/tazobactam | 16 | S |
| Cefazolin | ≥64 | R |
| Ceftazidime | 8 | S |
| Cefepime | 4 | S |
| Imipenem | 2 | S |
| Meropenem | 0.5 | S |
| Gentamicin | 2 | S |
| Tobramycin | ≤1 | S |
| Amikacin | ≤2 | S |
| Ciprofloxacin | ≤0.25 | S |
| Levofloxacin | 0.5 | S |

## Supplementary Table S3

**Phage annotation has functional proteins.**

| No. | Start position | Stop position | Nucleotide length | Prediction function | Similar species | Identified |
| --- | --- | --- | --- | --- | --- | --- |
| ORF1 | 1 | 375 | 375 | ATPase | Pseudomonas phage PaP1 | 100.00% |
| ORF2 | 385 | 1251 | 867 | ribose-phosphate pyrophosphokinase | Pseudomonas phage PaP1 | 100.00% |
| ORF4 | 1523 | 3211 | 1689 | Putative nictotinate phosphoribosyl transferase | Pseudomonas phage vB_PaeM_B31 | 99.82% |
| ORF6 | 3417 | 3896 | 480 | phosphatase | Pseudomonas phage C11 | 100.00% |
| ORF9 | 4367 | 4948 | 582 | DprA-like DNA recombination-mediator protein | Pseudomonas phage PaoP5 | 100.00% |
| ORF69 | 28157 | 29902 | 1746 | ribonucleotide reductase | Pseudomonas phage PaoP5 | 99.66% |
| ORF70 | 29895 | 30941 | 1047 | ribonucleotide reductase | Pseudomonas phage PaP1 | 99.71% |
| ORF71 | 30958 | 31302 | 345 | thymidylate synthase | Pseudomonas phage PaP1 | 100.00% |
| ORF72 | 31304 | 32269 | 966 | thymidylate synthase | Pseudomonas phage PaGz-1 | 100.00% |
| ORF74 | 32474 | 33424 | 951 | 3'-phosphatase, 5'-polynucleotide kinase | Pseudomonas phage vB_VIPPAEUMC01 | 99.37% |
| ORF79 | 34399 | 35178 | 780 | metallophosphoesterase | Pseudomonas phage PaP1 | 100.00% |
| ORF85 | 36579 | 37142 | 564 | HNH endonuclease | Pseudomonas phage vB_PaeM_B31 | 99.47% |
| ORF86 | 37139 | 38191 | 1053 | putative exodeoxyribonuclease | Pseudomonas phage PAK_P4 | 99.14% |
| ORF92 | 40775 | 41173 | 399 | NAD(P)H-dependent oxidoreductase | Pseudomonas phage SRT6 | 100.00% |
| ORF93 | 41263 | 41952 | 690 | DNA polymerase I | Pseudomonas phage K8 | 100.00% |
| ORF94 | 42237 | 44246 | 2010 | DNA polymerase | Pseudomonas phage K8 | 100.00% |
| ORF95 | 44307 | 46169 | 1863 | DNA primase/helicase | Pseudomonas phage JG004 | 100.00% |
| ORF105 | 48607 | 48996 | 390 | HAD-like domain protein | Pseudomonas phage SRT6 | 99.22% |
| ORF111 | ORF111 | 52098 | 1146 | RNA ligase | Pseudomonas phage vB_PaM_EPA1 | 99.74% |
| ORF113 | 52249 | 53058 | 810 | nucleoside triphosphate pyrophosphohydrolase | Pseudomonas phage vB_PaM_EPA1 | 98.88% |
| ORF118 | 54391 | 54522 | 132 | Rz-like spanin | Pseudomonas phage PAK_P2 | 100.00% |
| ORF119 | 54533 | 54976 | 444 | Rz-like spanin | Pseudomonas phage vB_PaeM_C2-10_Ab1 | 100.00% |
| ORF120 | 54963 | 55202 | 240 | holin | Pseudomonas phage PaP1 | 100.00% |
| ORF121 | 55220 | 55780 | 561 | endolysin | Pseudomonas phage PaP1 | 100.00% |
| ORF122 | 55797 | 57296 | 1500 | tail fiber protein | Pseudomonas phage PAK_P2 | 100.00% |
| ORF123 | 57308 | 57784 | 477 | tail assembly chaperone protein | Pseudomonas phage vB_PaeS_B8 | 100.00% |
| ORF124 | 57786 | 58166 | 381 | tail assembly chaperone | Pseudomonas phage vB_PaeS_B8 | 100.00% |
| ORF125 | 58202 | 60217 | 2016 | putative tail fiber protein | Pseudomonas phage vB_PaeM_B55 | 98.96%  ORF126 |
| ORF126 | 60228 | 60959 | 732 | structural protein | Pseudomonas phage JG004 | 100.00% |
| ORF127 | 60978 | 62441 | 1464 | baseplate wedge subunit | Pseudomonas phage PaP1 | 100.00% |
| ORF129 | 62825 | 63565 | 741 | baseplate spike | Pseudomonas phage PaP1 | 100.00% |
| ORF130 | 63562 | 64479 | 918 | baseplate hub | Pseudomonas phage PaP1 | 100.00% |
| ORF131 | 64476 | 64832 | 357 | virion structural protein | Pseudomonas phage PaP1 | 100.00% |
| ORF132 | 64838 | 65599 | 762 | tail fiber protein | Pseudomonas phage PaP1 | 100.00% |
| ORF133 | 65596 | 67962 | 2367 | tail length tape measure protein | Pseudomonas phage vB_VIPPAEUMC01 | 99.75% |
| ORF135 | 68219 | 68590 | 372 | tail assembly chaperone | Pseudomonas phage JG004 | 100.00% |
| ORF139 | 70213 | 71499 | 1287 | tail sheath | Pseudomonas phage K8 | 99.77% |
| ORF141 | 72072 | 72452 | 381 | head protein | Pseudomonas phage JG004 | 100.00% |
| ORF142 | 72452 | 72865 | 414 | putative RNA polymerase | Pseudomonas phage JG004 | 100.00% |
| ORF144 | 73429 | 74463 | 1035 | major head protein | Pseudomonas phage PAK_P1 | 100.00% |
| ORF147 | 75859 | 76329 | 471 | DNA methyltransferase | Pseudomonas phage JG004 | 100.00% |
| ORF148 | 76339 | 77778 | 1440 | portal protein | Pseudomonas phage vB_PaeM_C2-10_Ab1 | 100.00% |
| ORF149 | 77791 | 79311 | 1521 | terminase large subuni**t** | Pseudomonas phage JG004 | 100.00% |
| ORF155 | 83963 | 84511 | 549 | ATP-dependent protease | Pseudomonas phage JG004 | 100.00% |
| ORF165 | 86888 | 87304 | 417 | dCMP deaminase | Pseudomonas phage PAK_P1 | 100.00% |
| ORF166 | 87301 | 88509 | 1209 | putative DNA ligase | Pseudomonas phage vB_PaeM_B31 | 100.00% |
| ORF168 | 89048 | 89608 | 561 | cell wall hydrolyses involved in spore germination | Pseudomonas phage vB_PaeM_LCK69 | 98.92% |
| ORF169 | 89610 | 90170 | 561 | metal-dependent phosphohydrolase | Pseudomonas phage PaP1 | 100.00% |
| ORF171 | 90591 | 91145 | 555 | phosphoesterase | Pseudomonas phage vB_PaeM_C2-10_Ab1 | 100.00% |
| ORF173 | 91395 | 91670 | 276 | HTH DNA binding protein | Pseudomonas phage C11 | 100.00% |
| ORF174 | 91667 | 92074 | 408 | membrane protein | Pseudomonas phage C11 | 100.00% |
| ORF175 | 92086 | 93003 | 918 | RNA ligase and tail fiber protein attachment catalyst | Pseudomonas phage PAK_P2 | 100.00% |
| ORF176 | 93014 | 93181 | 168 | ATPase | Pseudomonas phage vB_PaeM_MAG1 | 100.00% |
